# Efficacy of MTAD, Glyde and EDTA in debridement of curved root canals

**Published:** 2009-04-17

**Authors:** Nahid Mohammadzadeh Akhlaghi, Elahe Behrooz, Mohammad Ali Saghiri

**Affiliations:** 1 *Department of Endodontics, Dental School, Islamic Azad University of Medical Sciences, Tehran, Iran.*; 2 *Dentist, Islamic Azad University of Medical Sciences, Tehran, Iran.*; 3 *Master of Science in Biomaterials, PhD Student of Michigan University, Michigan, USA.*

**Keywords:** Debris, EDTA, Glyde, MTAD, Smear layer

## Abstract

**INTRODUCTION:** This study aimed to compare the efficacy of MTAD, Glyde and EDTA in removing the post-preparation smear layer and debris produced in apical third of curved molar root canals.

**MATERIALS AND METHODS**: Forty extracted human maxillary first molars with curved mesiobuccal canals (30˚-35˚), similar root lengths and morphologies were divided into 3 experimental groups (n=12) and one control group (n=4). Canals were prepared by RaCe rotary files and were treated with the following materials between each two files: Group A (control group); 5 mL of distilled water, Group B; 1 mL of 17% ethylenediamine tetra-acetic acid (EDTA) for 1 min, Group C; BioPure^TM^ MTAD (according to the manufacturer’s instruction; 5mL for 5 min), and Group D; Glyde File Prep alternated with sodium hypochlorite (NaOCl) between each two files. Debridement of the apical third was evaluated using scanning electron microscope (SEM) (×5000). The data were statistically analyzed using the Kruskal-Wallis and Mann-Whitney U tests (P<0.05).

**RESULTS:** Statistical analysis of the data showed that MTAD and 17% EDTA were effective in removing smear layer and debris from the apical thirds. MTAD was more effective than EDTA in smear layer removal. Glyde did not adequately debride curved root canals of molar teeth.

**CONCLUSION:** MTAD and 17% EDTA were effective in debriding the apical third of curved molar root canals during endodontic treatment, whereas Glyde File Prep does not provide adequate debridement.

## INTRODUCTION

Mechanical instrumentation of root canals produces a smear layer composed of organic and inorganic substances such as dentin particles, necrotic debris, microorganisms and odontoblastic processes (1). Despite the controversies regarding smear layer (2), most clinicians have concluded that the removal of smear layer is beneficial as it harbors microorganisms (3), reduces dentin perme-ability and prevents the contact of irrigants with dentinal tubules (4), compromises adaptation of obturation materials to root surfaces, and hinders sealer penetration into dentinal tubules (5). Although a recent study showed no difference between leakage through smear-free and smear-covered dentin (6); among the 65 studies that evaluated the effects of the smear layer on the seal of obturated root canals, 35 (53.8%) have reported no significant difference between smear-free and smear-covered root canal surfaces, 27 (41.5%) have reported results in favor of smear layer removal and 3 (4.7%) are in favor of keeping the smear layer. The general consensus is that the smear layer has adverse affects on root canal seal (7,8).

Presently, irrigation is the best method to remove tissue remnants and dentin debris during instrumentation (9). To achieve simultaneous removal of organic tissue and smear layer, combined use of NaOCl and other irrigants such as chelating agents are recommended (10).

BioPure^TM^ MTAD (Densply Tulsa Dental, Tulsa, OK) is a mixture of a tetracycline isomer (Doxycycline), an acid (citric acid) and a detergent (Tween^®^ 80), which was introduced by Torabinejad *et al.* in 2003 (11). MTAD removes the smear layer safely and completely (11), disinfects the root canal efficiently (12) and is also biocompatible (13). A recent survey evaluating the current use of new endodontic technology and materials by diplomats of the American Board of Endodontists revealed the wide use of this irrigant during endodontic treatments and retreatments. In retreatment cases with closed apices, MTAD has been the irrigant of choice (14).

Glyde^TM^ File Prep (Dentsply Maillefer, Ballaigues, Switzerland) is a root canal conditioner consisting of EDTA and carbamide peroxide in a water-soluble base. According to the manufacturer’s instructions, this gel is capable of removing the smear layer due to its EDTA content (10,15).

17% EDTA (ethylenediamine tetra-acetic acid) is a chelating agent capable of removing inorganic material and the smear layer (10). Several investigations have shown that the use of 17% EDTA can cause inadvertent dentinal wall erosion (11,16,17).

Different studies have assessed the efficacy of these materials in debridement of straight canals in single-rooted teeth (10,11,15,16,18-21). There are no studies evaluating their efficacy in reaching and debriding the apical third of curved root canals. The objective of this study was to compare MTAD, Glyde and 17% EDTA’s effectiveness in curved mesiobuccal canals of upper first molars.

## MATERIALS AND METHODS

Forty virgin healthy extracted human maxillary first molars with mesiobuccal canal curvatures ranging between 30˚-35˚ (according to Schneider’s method (22)) with similar root lengths were selected for this study. After access cavity preparation, the working lengths were determined K-file size #10 (Dentsply Maillefer, Ballaigues, Switzerland). Samples were randomly divided into 4 groups A, B, C (n=12) and D (n=4); A being the positive control group. After coding the samples, mesiobuccal canals were instrumented using RaCe rotary files (FKG, Dentaire, La chaux-de-fonds, Switzerland) in the following crown-down sequence: #40 (0.1), #35 (0.08), #30 (0.06), #25 (0.06). A size #30 (0.06) was used as the master apical file (MAF) in all the samples. In group D, Glyde File Prep and NaOCl were used alternately between each instrument according to the manufacturer’s instruction. The specimens in groups A, B and C were irrigated with 1 mL of 5.25% NaOCl (Samen Pharmaceuticals, Mashhad, Iran) between each file using a 28-gauge needle (DENTSPLY Tulsa Dental, Tulsa, OK) and were finally irrigated 1-1.5 mm short of the working lengths with one of the following solutions:

Group A: (positive control): 5 mL of distilled water; Group B: 1 mL of 17% EDTA (Asia Chemi Teb Mfg, Tehran, Iran) for 1 minute; Group C: 5 mL of BioPure MTAD for 5 minutes. According to the manufacturer’s instruction MTAD should be freshly prepared before final irrigation of the root canals. As recommended by the manufacturer, 5 mL dosages were used for each canal. Content of the 5 mL syringes was gradually injected into the powder bottle and the combination was gently shaken for 60 seconds with the syringe attached to the bottle. Once completely mixed the solution was drawn into the 5 mL delivery syringes, the syringe was removed from the bottle and after attaching the 28 gauge needle the canals were irrigated as follows: the needle was passively placed into the canal space, 1-1.5 mm short of working length and 1 mL of the solution was slowly injected into the canal. A #15 cotton-wrapped barbed broach was placed to working length and left inside the canal space for 4 min. Subsequently the barbed broach was removed and the solution suctioned. The canal was rinsed with the remaining 4 mL of the solution for 1 min (total irrigation time of 5 min).

**Figure 1:**
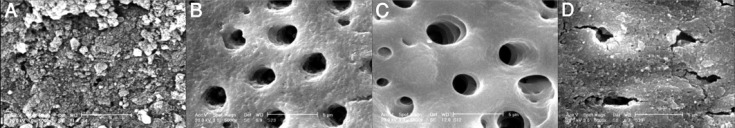
SEM of the dentinal surfaces in the apical third of the samples (original magnification ×5,000). (A) control group. (B) 17% EDTA-treated sample. (C) MTAD-treated sample. (D) Glyde-treated sample

**Table 1:** The percentage of frequency for smear layer and debris removal in the four groups

**Groups removal** **Scores**	**Debris removal**					**Smear layer**				
	**1**	**2**	**3**	**4**	**5**	**1**	**2**	**3**	**4**	**5**
**EDTA (n=12)**	83.3	16.7	0	0	0	25	75	0	0	0
**MTAD (n=12)**	100	0	0	0	0	91.7	8.3	0	0	0
**Glyde (n=12)**	0	58.3	25	16.7	0	0	0	16.7	83.3	0
**Control (n=4)**	0	0	25	75	0	0	0	0	100	0

Before SEM preparation, all samples were irrigated with 10 mL of distilled water to remove any remnants of the final irrigant and the apical thirds were defined by 2 grooves on the buccal and lingual surfaces to facilitate SEM analysis. The mesiobuccal roots were separated; vertically grooved on the buccal and lingual surfaces with a diamond disc without transgressing into the canals, and split longitudinally. Half of each sample was randomly chosen, placed in 2% glutaraldehyde for 24 hours and then rinsed 3 times with a sodium cacodylate buffered solution (0.1 M, pH 7.2). After incubation in osmium tetroxide for 1 hour, the samples were dessicated with ascending concentrations of ethyl alcohol (30-100%), placed in a desicator for 24 hours and mounted on a metallic stub. After coating the samples with 20µ of gold, SEM photomicro-graphs were taken using a back scatter mode (XL30, Philips, Holland) (×5000). Three observers scored the amount of smear layer and debris in a blind manner according to Schäfer’s grading criteria (23). Scores 1 and 2 represented acceptable debridement; scores 3, 4 and 5 represented unacceptable debridements. The data were statistically analyzed using the Kruskal-Wallis and Mann-Whitney *U* tests (P<0.05).

## RESULTS

The Kruskal-Wallis test showed significant statistical difference between the four groups (P<0.05) (Table 1). All samples in the control group were covered with smear layer and debris (Figure 1A). All MTAD and EDTA treated samples showed acceptable debridement (scores 1 and 2) (Figure 1B, Figure 1C). They had no significant difference in debris removal (P=0.148), but with regard to smear layer removal, MTAD was significantly more effective (P=0.028). While 91.7% of the MTAD treated samples received score 1 (Table 1), only one sample (8.3%) received score 2. Twenty five percent of the EDTA-treated samples were scored 1 and the rest (75%) received score 2. The Mann-Whitney *U* test showed that the Glyde group had significantly better debris removal than the control group (P=0.020), however, both groups showed unacceptable smear layer removal (P=0.398) (Figure 1D).

## DISCUSSION

Many investigations have assessed the efficacy of MTAD, Glyde and EDTA independently in removing the smear layer and debris in straight canals of single-rooted teeth (1,10,11,15,18-21,24). Arguably, in curved root canals (25) and canals with inadequate apical preparations (26), irrigants are likely to be less effective.

This study revealed that MTAD and EDTA are effective in smear layer and debris removal; whereas Glyde and NaOCl do not efficiently debride the apical thirds of curved root canals. Because of the delicate apical regions of upper first molars, MAF sizes of previous similar studies (11,19,27), and a report of adequate penetration of irrigants in root canals with apical preparations up to a file #30 (0.06) (28), we chose a file #30 (0.06) as MAF in all samples. Our results concur with Khademi *et al.* (28), who showed adequate cleaning in the apical portion with this file size.

Our study provides further evidence that NaOCl alone is ineffective in smear layer and debris removal (10,11,19). 

Glyde also failed to adequately remove smear layer in the apical region in this study as well as others (15,29). Lim *et al.* (10) reported no significant difference between the effect of Glyde and EDTA in the apical third of canals. Both were more effective than NaOCl, with significant superiority in the coronal and middle thirds. They used single-rooted teeth with less root curvature (<30˚) and higher MAF sizes, which facilitated the penetration of debriding substances to the apical region and resulted in better smear layer removal with Glyde.

Grandini *et al.* (20) used straight-rooted anterior teeth reported Glyde to be more effective than NaOCl in removing debris, but unable to eliminate debris or smear layer in the apical third of root canals; concurring with our study. Apical thirds of root canals are smaller in diameter; therefore, efficient penetration of irrigants is difficult (15,19,30).

Similar to previous studies (16,19,24), we used 1 mL of 17% EDTA for 1 minute as a final rinse. Despite the curvature and delicate nature of our samples, we found effective smear layer removal in the apical region (11,31-33). Pérez-Heredia *et al.* also showed that acidic solutions such as 17% EDTA improve smear layer removal when used in conjunction with NaOCl (21). Our results corroborate with the findings of Crumpton *et al.* which demonstrated complete removal of smear layer and lower erosiveness of this irrigation regimen (24). Numerous researchers have shown the erosive effects of EDTA when applied in higher volumes and longer times (1,11,16,17,34). Calt *et al.* showed that the application of 17% EDTA for over 1 minute can cause adverse erosion of the root canal surfaces (16).

Khedmat and Shokouhinejad reported that 1 mL of chelating agents, including 17% EDTA, was unable to eliminate the smear layer from the apical third of canals in 1 minute, but showed better results than those irrigated with NaOCl alone (19). Despite using single-rooted teeth, 17% EDTA displayed better results in this study with curved canals.

MTAD treated samples showed ideal smear layer and debris removal in our study. These results confirm the findings of previous investigations (11,27,31,32). In this study MTAD was superior to EDTA in removing smear layer. Torabinejad *et al.* used single-rooted teeth or the largest canals in multi-rooted teeth, with file #30 (0.04) as the MAF (11,27). They reported smear-free dentinal walls in MTAD-treated samples. They detected no significant difference between 17% EDTA and MTAD with regard to smear layer removal in the coronal and middle thirds of the canals, but reported MTAD to be superior in removing debris from the apical third of the root canals (11). They, however, used higher volumes of 17% EDTA for longer periods of time.

Tay *et al.* showed similar results *i.e.* complete smear layer removal after a final rinse of MTAD (according to the manufacturer’s instruction) (31,32). They irrigated the samples with 30-guage needles with 5 mL of 17% EDTA for 2 and 5 minutes, and reported that both MTAD and 17% EDTA completely eliminated the smear layer.

## CONCLUSIONS

Based on the results of this study, irrigating the apical portions of curved root canals with MTAD resulted in complete smear layer and debris removal. Final irrigation of such samples with 1 mL of 17% EDTA for 1 minute removed the smear layer and debris effectively, but did not result in ideal smear layer removal. Application of Glyde removed some of the debris, however it was unable to remove the smear layer.
